# Quantitative Pharmaco-Electroencephalography in Antiepileptic Drug Research

**DOI:** 10.1007/s40263-018-0557-x

**Published:** 2018-08-27

**Authors:** Yvonne Höller, Christoph Helmstaedter, Klaus Lehnertz

**Affiliations:** 1 Department of Neurology, Christian Doppler Medical Centre and Centre for Cognitive Neuroscience, Paracelsus Medical University, Ignaz Harrer Str. 79, 5020 Salzburg, Austria; 2 Present Address: Department of Psychology, University of Akureyri, Norðurslóð 2, 600 Akureyri, Iceland; 3 Department of Epileptology, University of Bonn, Sigmund Freud Straße 25, 53105 Bonn, Germany; 4 Interdisciplinary Center for Complex Systems, University of Bonn, Brühler Straße 7, 53175 Bonn, Germany

## Abstract

Pharmaco-electroencephalography (pharmaco-EEG) has never gained great popularity in epilepsy research. Nevertheless, the electroencephalogram (EEG) is the most important neurological examination technique in this patient population. Development and investigation of antiepileptic drugs (AEDs) involves EEG for diagnosis and outcome evaluation. In contrast to the common use of the EEG for documenting the effect of AEDs on the presence of interictal epileptiform activities or seizures, quantitative analysis of drug responses in the EEG are not yet standard in pharmacological studies. We provide an overview of dedicated pharmaco-EEG studies with AEDs in humans. A systematic search in PubMed yielded 43 articles, which were reviewed for their relevance. After excluding studies according to our exclusion criteria, nine studies remained. These studies plus the retrieved references from the bibliographies of the identified studies yielded 37 studies to be included in the review. The most prominent method in pharmaco-EEG research for AEDs was analysis of the frequency content in response to drug intake, often with quantitative methods such as spectral analysis. Despite documenting the effect of the drug on brain activity, some studies were conducted in order to document treatment response, detect neurotoxic effects, and measure reversibility of AED-induced changes. There were some attempts to predict treatment response or side effects. We suggest that pharmaco-EEG deserves more attention in AED research, specifically because the newest drugs and techniques have not yet been subject to investigation.

## Key Points

**Table:** 

The most prominent method in pharmaco-electroencephalography (pharmaco-EEG) research for antiepileptic drugs (AEDs) is analysis of the frequency content in brain response to drug intake.
Pharmaco-EEG deserves more attention in AED research, specifically because the newest drugs and techniques have not been subject to investigation to date.

## Introduction

The electroencephalogram (EEG) is a standard assessment tool in neurological evaluations and for documentation of functional activity changes. EEG biomarkers can be extracted from a resting EEG or an EEG under cognitive or sensory stimulation, both with or without administration of drugs. Quantitative analysis of the EEG in order to document the effect of drugs on electric activity of the brain is known as pharmaco-EEG. According to the pharmaco-EEG guidelines of the International Pharmaco-EEG Society reported by Jobert et al. in 2012 [1], “pharmaco-EEG concerns the description and the quantitative analysis of the effects of substances on the central nervous system (CNS) by means of neurophysiological and electrophysiological methods used within the framework of clinical and experimental pharmacology, neurotoxicology, therapeutic research and associated disciplines”. Pharmaco-EEG is nearly as old as the EEG itself, since it was Hans Berger himself who first examined the effects of different substances on the EEG [2]. Even the use of Fourier analysis as a quantitative means for characterizing the EEG can be dated back to Berger in 1932 [3].

The effect of drugs on the EEG has been emphasized in several publications on clinical applications of psychiatric medication [4–15], and thus EEG analysis has become the basis for the classification of new drugs, especially in psychopharmacology [16–19]. It has been claimed that pharmaco-EEG is non-invasive, bears no risks for the subjects, and is inexpensive and highly available [19]. The International Pharmaco-EEG Society has provided guidelines in order to standardize and stimulate pharmaco-EEG studies in human subjects [1]. However, the potential of pharmaco-EEG is yet to be fully exploited.

The EEG is a standard assessment tool and, despite the considerable hype around imaging techniques, we believe it still might be the most important one in epilepsy. In contrast to the many studies in psychopharmacology, the use of pharmaco-EEG in epilepsy has only rarely been the subject of studies explicitly identified as pharmaco-EEG research. Nevertheless, pharmaco-EEG is actually an everyday practice in the diagnosis and treatment of epilepsy. The EEG delivers essential information regarding whether interictal epileptiform activities exist in patients reporting seizure-like events. After initiating treatment, the EEG can be used to monitor the response to the drug, for example whether the presence of the interictal epileptiform activities has changed [20].

Despite these common applications of the EEG for the evaluation of the effectiveness of antiepileptic drugs (AEDs), there is currently no active pharmaco-EEG research in epilepsy being undertaken. Jobert et al. [1] summarized the reasons for this lack of popularity of pharmaco-EEG in clinical research and practice, which included low acceptance of the EEG as a biomarker, variance of EEG responses across drugs, a lack of standards for operating procedures, large inter-individual variability in EEG records, and, as a historic reason, the large storage and processing capabilities that are needed to record EEG signals. However, the authors note that the technical limitations are no longer of relevance. We would like to extend this list by emphasizing that one major obstacle may be technical difficulties that prevent clinical staff from integrating pharmaco-EEG into daily routine. So far, no normative information is integrated into standard clinical EEG software, and no user-friendly interface is available that could aid the quantitative interpretation of neurophysiological effects of AEDs.

Despite these obstacles, we are convinced that estimation of the potential of pharmaco-EEG for epilepsy is highly warranted. Therefore, we aim to provide an overview of the research that has been conducted so far on pharmaco-EEG in epilepsy. The present review should allow characterization of the potential of pharmaco-EEG for AEDs and stimulate new research questions.

## Literature Search Methodology

We performed a systematic search of the literature in PubMed (accessed on 22 February 2018) with the following search terms:

- pharmaco-EEG AND epilepsy (yielding eight articles)
- pharmaco-EEG AND antiepileptic AND drug (yielding 43 articles).

The eight articles from the first search were all included in the 43 articles from the second search. We excluded articles according to the following criteria:

- Two studies: available in Russian only.
- Two studies: neither the abstract nor full text was retrievable.
- Seven studies: conducted in animals (rabbits, mice, or rats).
- Twenty-three studies: targeted at other drugs such as tranquilizers and were not conducted in epilepsy patients.

Animal studies are not uncommon in drug research, and even pharmaco-EEG is conducted in animals. However, for the purpose of this review we excluded these studies as our aim was to summarize AED effects in the human EEG. For a comprehensive overview of pharmaco-EEG studies in animals see the recent review by Drinkenburg et al. [21].

We did not exclude studies that used provocation methods, as the provocation (e.g., by photic stimulation) is part of standard routines in clinical EEG and should also be combined with pharmaco-EEG.

The references of the remaining articles were reviewed for further studies. The nine remaining studies plus the retrieved references yielded 37 studies to be included in the review.

## Early Applications of Pharmaco-Electroencephalography (Pharmaco-EEG) for Antiepileptic Drug (AED) Evaluation

The main endpoint in most pharmaco-EEG studies involving AEDs was assessment of the frequency characteristics, either visually or by quantitative measures. It has been stated that the application of quantitative EEG in the assessment of AEDs and drugs for the treatment of psychiatric conditions aims to understand the effects in the central nervous system (CNS) [22]. Thus, the research in AEDs and psychiatric drugs does not differ with respect to the intent. Early studies [23–26] critically evaluated the effects of AEDs on the EEG. In early 1978, Rosadini and Sannita [22] criticized the inconclusive nature of non-quantitative or semi-quantitative studies about effects of AEDs on background EEG activity; the authors claimed to be the first to apply quantitative measures. Their study reported a very detailed analysis of spectral power in repeated EEG recordings over up to 16 months of treatment, which were assessed together with plasma concentration in ethosuximide, diphenylhydantoin, valproic acid, and phenobarbital.

As a result of intensive research during this period, in 1982 Schmidt summarized 23 controlled trials with therapeutic drug monitoring and repeated EEG recordings [27]. The main conclusions focused on AED plasma concentration-related suppression of paroxysmal discharges (diazepam, phenobarbital, and phenytoin), increase in beta (β) activity (clonazepam, phenytoin, and phenobarbital), slowing of background activity (diazepam and phenytoin), clinical seizure frequency in relation to paroxysmal discharges (phenobarbital and phenytoin), and drug toxicity in relation to EEG slowing (carbamazepine, phenytoin, phenobarbital, and primidone).

It was soon acknowledged that short-term administration under EEG control and pharmaco-EEG studies have become an important source of information about the clinical effects of AEDs [28]. Despite the focus of most publications being on pharmaco-EEG spectral characteristics, in 1988 Scollo-Lavizzari et al. [28] emphasized reduced spike and wave counts in relation to the short-term effects of flumazenil. However, in a later review [29] it was concluded that the relation between interictal epileptiform activity and seizure control is somewhat arbitrary. In addition to frequency responses and epileptiform activity, event-related potentials can be used to assess AEDs, for example in terms of neurotoxicity. Lamotrigine seems to be less neurotoxic than phenytoin on the basis of an increased latency in the brainstem auditory-evoked response [30].

In summary, examination of epileptiform activity and event-related potentials was part of pharmaco-EEG research, but frequency analysis was and is the most prominent tool.

## Frequency Responses to AEDs

In the 1980 s and 1990s, several studies continued the examination of EEG frequency responses to AEDs. As is evident in Table 1, the most common findings are EEG slowing, being reflected by an increase of delta (*δ*; 0–4 Hz) and theta (*θ*; 5–7 Hz) activity and a decrease of activity in higher frequency ranges, and specifically slowing of the dominant rhythm [29]. Most importantly, these drug-related changes are reversible even under short-term drug withdrawal [31–33].

**Table 1:** Overview of frequency responses to antiepileptic drugs

Drug	Mechanism	Frequency effect	Sample	Study (year)
Ethosuximide	Calcium channels: blockade of low voltage-activated channel	– *δ*, + *α*	6 patients	Rosadini and Sannita (1978) [22]
Diphenylhydantoin	Sodium channels: blockade by stabilizing fast-inactivated state	Nothing	5 patients	Rosadini and Sannita (1978) [22]
Carbamazepine	Sodium channels: blockade by stabilizing fast-inactivated state	+ *δθ*	45 uncontrolled partial and generalized epilepsy patients	Wilkus et al. (1978) [26]
		+ General, + *δθ*, – *α*	10 untreated patients	Besser et al. (1992) [42]
		Mild slowing	15 healthy subjects	Meador et al. (1993) [43]
		+ *δθ*, slowing	31 healthy, 6 patients	Salinsky et al. (1994) [39]
		– PF	16 untreated children	Frost et al. (1995) [71]
		+ *θ*, – u*α*	10 healthy, 10 patients	Wu and Xiao (1996) [38]
		+ *δθ*, – PF	11 healthy	Salinsky et al. (2002) [37]
		+ *δθ*, – α	20 difficult-to-treat partial epilepsy patients	Clemens et al. (2004) [41]
		+ *θ*, – PF	41 untreated patients	Clemens et al. (2006) [40]
		+ *θ*, – *α*, + P3 latency	60 healthy	Meador et al. (2016) [55]
Phenytoin	Sodium channels: blockade by stabilizing fast-inactivated state	– *δθ*, + *αβ*	12 healthy males	Fink et al. (1979) [85]
		+ *δθ*	27 patients	Herkes et al. (1993) [44]
		Mild slowing	15 healthy subjects	Meador et al. (1993) [43]
		– *α*	7 healthy	Chung et al. (2002) [52]
Clonazepam	Benzodiazepine; activation of GABA_A_ receptor	+ *β*	21 children with epilepsy	Dumermuth et al. (1983) [67]
		+ Fast activity	4 children with epilepsy	Wang and Wang (2002) [49]
Milazemide	Increasing glycine concentrations	– *δ*, + *α*, + PF	12 healthy	Saletu and Grünberger (1984) [34]
Gabapentin	Calcium channels: blockade of high voltage activated channel	– General, + *δθ*, – *α*	10 healthy	Saletu et al., (1986) [72]
		+ *δ*θ, – PF	12 healthy	Salinsky et al. (2002) [37]
Valproate	Synaptic vesicle protein 2A actions	– General, – uαβγ	10 untreated patients	Sannita et al. (1989) [35]
		+ Power of PF	12 patients, 12 healthy	Wu and Ma (1993) [48]
		+ General, – u*αβ*	12 patients	Sannita et al. (1993) [45]
		+ u*α*, – *β*	10 untreated patients	Wu and Xiao (1997) [46]
		Nothing	4 long-term treated children	Wang and Wang (2002) [49]
		– *δθα*	42 untreated patients	Clemens et al. (2006) [40]
Oxcarbazepine	Sodium channels: blockade by stabilizing fast-inactivated state	– PF	9 untreated patients	Clemens et al. (2006) [40]
Lamotrigine	Sodium channels: blockade by stabilizing fast-inactivated state	– *δθαβ*, +PF	25 untreated patients	Clemens et al. (2006) [40]
Lacosamide	Sodium channels: blockade by stabilizing slow-inactivated state	+ *θ*, – *α*	41 healthy	Meador et al. (2016) [55]

Drugs are ordered by chronological order of pharmaco-electroencephalography research conducted with the respective drug

Frequency ranges are according to the definitions in the referenced articles

*α* alpha 8–12 Hz, *β* beta 13–30 Hz, *γ* gamma > 30 Hz, *δ* delta ≤ 4 Hz, *θ* theta 5–7 Hz, *GABA* γ-aminobutyric acid, *general* broadband, *nothing* no effects detected, *PF* peak frequency, *uα* upper α 10–13 Hz, + indicates increase in activity, – indicates decrease in activity

In contrast to most other agents, milacemide was reported to rather accelerate the EEG. One study examined milacemide using pharmaco-EEG in healthy subjects [34]. The agent increases *γ*-aminobutyric acid (GABA) concentrations and endogenous glycine pools. It was shown that δ activity was reduced, slow rhythms were accelerated, and *β* (14–30 Hz) and alpha (*α*; 8–13 Hz) activity were increased depending on the dosage. These effects were interpreted as being indicative of improvement of vigilance at low doses and might be linked to the mechanism of action, which differs from the majority of the other drugs. Milacemide is an activating rather than an inhibiting drug, and the EEG reflects this property very well.

However, the most typical finding for several drugs is the overall attenuation of power, such as in response to sodium valproate [35] and gabapentin [36]. Drugs acting via blockade of calcium or sodium channels exhibit slowing effects in the EEG. For example, gabapentin was found to augment *δ* and *θ* activity and to decrease *α* power in healthy participants [36, 37]. Despite these initial slowing effects, there might be beneficial effects on alertness, as these are emergent some hours after drug administration [36]. These results suggest that examination of the frequency effects of drugs should be followed up over several hours, or even over months [22].

According to Table 1, carbamazepine is the best examined AED in terms of pharmaco-EEG. By correlating the EEG changes with the serum concentration of carbamazepine, a progressive increase of *θ* power and decrease of upper *α* power (10–13 Hz) was shown [38]. The effects of carbamazepine documented by earlier studies [38, 39] were largely replicated in a later study [40]. The authors of this later study observed that carbamazepine increased *θ* activity, carbamazepine and oxcarbazepine decreased mean *α* frequency, lamotrigine increased mean α frequency, and both lamotrigine and valproate reduced broadband power. In patients switching from carbamazepine to oxcarbazepine it was reported that, under oxcarbazepine, the power of *δ*, *θ*, and *α* bands was reduced, while β activity was increased in relation to the effect that was observed under carbamazepine [41]. Indeed, such an EEG slowing in terms of increased δ and/or θ power and decrease of the dominant posterior rhythm seems to be typical for carbamazepine [39, 42, 43] and phenytoin [39, 44]. In addition, memory performance was impaired under medication with carbamazepine or phenytoin [43].

A decrease of β power with valproate is well-documented [45, 46]. Upper-α power on occipital areas increased with the serum concentration of valproate while *β* power over posterior scalp areas decreased [46]. As such, valproate was documented to normalize the EEG power spectrum in idiopathic generalized epilepsy [47]. However, valproate has a similar effect on patients as on healthy adults [48], by raising the power of the dominant frequency. It is important to note that after long-term therapy there is no correlation between the concentration of valproate in the blood and activity in the classical frequency bands [49].

Analogously to the effect of valproate, alterations of β activity were also associated with diazepam [50]. The percentage of diazepam-induced change in *β* activity was reduced in patients with epilepsy compared with healthy controls, and especially in patients with long-term AED treatment, refractory epilepsy, and brain structural damage.

Therefore, it is necessary to take the patient’s medical history into account. In addition, it was noted that the effects of AEDs on EEG background rhythms vary considerably between patients and it is necessary to test the stability of responses over time [39]. A 1-year long-term study documented the course of EEG changes under antiepileptic medication in children; the authors observed a normalization of pathologic slowing [51]. However, this long-term effect differed between patient populations and treatment. Thus, for the assessment of AED effects on the EEG, several factors need to be taken into account, such as age of the patient, medication history, severity of epilepsy, type of AED and mechanism of action, dosage, structural damage to the CNS, and duration of therapy.

Table 2 summarizes the results in terms of the frequency ranges as defined by the International Pharmaco-EEG Society [1]. It should be noted that these ranges were not always used in the summarized publications, so in some cases low precision must be taken into account. The table demonstrates, again, that for most AEDs a slowing effect in the sense of an increase in slow activity below 6 Hz and a decrease in fast activity above 6 Hz is typical.

**Table 2:** Frequency effects of antiepileptic drugs as listed in Table 1, summarized for frequency ranges as pre-defined by the International Pharmaco-EEG Society [1]

Antiepileptic drug	Frequency range								
	1.5 to < 6.0	6.0 to < 8.5	8.5 to < 10.5	10.5 to < 12.5	12.5 to < 18.5	18.5 to < 21.0	21.0 to < 30.0	30.0 to < 40.0	DF^a^
Ethosuximide	–		+	+					
Diphenylhydantoin									
Clonazepam					+(+)	+(+)	+(+)	(+)	
Milazemide	–		+	+					+
Gabapentin	++	++	–	–					–
Valproate	–	–	–	– – –+	– –+	– – –	– – –	–	+
Carbamazepine	+++++	++++++++	– – –	– – – –					– – –
Phenytoin	–+	–+	+	+–	+–	+			
Oxcarbazepine									–
Lamotrigine	–	–	–	–	–	–	–		+
Lacosamide		+	–	–					

Drugs are ordered by chronological order of pharmaco-electroencephalography research

The number of signs (∓) indicates the number of studies that reported such an effect

Signs in parentheses indicate that the respective reference was not clear on the exact frequency range

*DF* dominant frequency, – indicates power decrease, + indicates power increase

^a^DF according to Jobert et al. [1]: 6.0 to < 12.5

## Pharmaco-EEG and Cognitive Side Effects

Another very important aspect is that AEDs affect cognitive functions, such as memory, attention and vigilance, and these changes are directly related to drug effects in the EEG. The cognitive effects are understood as side effects, be it an improvement or decline of functioning, since these changes are secondary to the intended purpose of seizure control.

An improvement in attention, concentration, psychomotor activity, and after-effect according to the Archimedean spiral was observed by Saletu and Grünberger [34] after low doses of milacemide (400 mg). However, with increasing dose, this positive effect declined. Similarly, *β* activity increased after 400 mg of milacemide but declined after 1600 mg. In a similar study, Saletu and co-workers [36] examined the effects of gabapentin in healthy subjects. As an immediate effect, *δ* and *θ* activity increased after 2 h while α activity decreased, indicating an inhibition of cognitive functions. After several hours, the changes reversed direction and were interpreted as being beneficial for vigilance. Psychometric assessment confirmed improvement in concentration, numerical memory, and reaction. Furthermore, for gabapentin and carbamazepine, the decrease in the peak frequency of the posterior α rhythm—a strong indicator for EEG slowing—coincided with an objective and subjective decrease of cognitive performance after 12 weeks in healthy volunteers [37]. A similar slowing effect in the EEG was observed by Meador et al. [43] when assessing the EEG effects of carbamazepine and phenytoin. Slowing effects in the EEG coincided with impaired memory performance in a verbal memory task.

In contrast, at low doses of phenytoin 10 mg/kg, *α* band power decrease and task-related attenuation of frontal midline *θ* responsivity and event-related potential modulations were detected in seven healthy volunteers in the absence of behavioral changes in a spatial working memory task [52]. However, the participants reported subjective effects of the drug, suggesting that compensatory effects might obscure the direct relation between EEG alterations and the behavioral effects of phenytoin at low doses. It was therefore suggested that pharmaco-EEG could be a means to find objective correlates of subjective reports of neurotoxicity [53].

An innovative approach was recently reported by Meador et al. [54], where a composite score was built from EEG properties and neuropsychological measures in order to describe the effects of brivaracetam and levetiracetam. The EEG effects were separated from the cognitive profile in a later study from the same group [55], where an increased P3 latency was observed with treatment with carbamazepine and lacosamide. The authors have performed multiple cognitive tests alongside the EEG and also formed a composite score. The evaluation of EEG effects of drug during cognitive performance is a very plausible approach to document the physiological correlates of cognitive side effects.

Taken together, the coincidence of pharmaco-EEG effects and cognitive alterations suggests that these are two complementary views of the same phenomenon.

A limitation of these studies that needs to be considered is that they were performed with healthy volunteers, while the effects could interact with epileptic aspects in patients with epilepsy. For example, we could hypothesize that the decrease in cognitive performance might be counterbalanced by the control of interictal epileptic activity and seizures, which might in turn negatively affect cognitive functioning.

However, Salinsky and co-workers [53] demonstrated that cognitive changes in patients with epilepsy starting or ending a therapy with AEDs correlated linearly with the peak frequency of the posterior rhythm (*r*^2^ = 0.71), including a digit symbol test, finger tapping, reading colors in a Stroop task, and a visual reaction task. This study represents very strong evidence for the direct relation between neurophysiological and neuropsychological drug effects.

## Prognosis of Treatment Response

Saletu et al. [56] anticipated that pharmaco-EEG—specifically topographic pharmaco-EEG imaging—could “(eventually) predict therapeutic efficacy in patients”. Starting a therapy with AEDs, conversion between monotherapy, or initiating polytherapy comes along with at least two questions: (i) will the new therapy help to control seizures? and (ii) will the new drug cause undesired or desired side effects? After several weeks of treatment, the new AED may turn out to have an insufficient effect on seizures, meaning that treatment conversion or additional treatment is needed. Adverse effects of AEDs, often described as a correlate of neurotoxicity, should ideally be recognized objectively at the beginning of treatment [40]. If we could predict the efficacy of treatment and side effects by giving the patient a single dose, we could avoid potentially harmful and expensive administration of drugs over months and—ideally—immediately find out the most efficient treatment strategy. Such a prognosis may be based on some kind of biomarker [57]. So far, research on predictability of therapeutic success or therapy refractoriness is mostly limited to imaging [58–60], with a clear focus on prognosis of therapy refractoriness. Quantitative EEG has been found to predict response to psychiatric medication [61–63] and to predict response to rivastigmine for the treatment of Alzheimer’s disease [64], and is of interest also for the prediction of AED response [57]. The current guidelines for pharmaco-EEG support the view that pharmaco-EEG is applicable whenever a drug is supposed to have an effect on the CNS [1]. While several detailed studies have addressed the EEG effects of treatment response in AEDs, we could identify only a minor number of studies examining the use of the EEG as a possible predictor of desired and undesired effects.

In the 1990 s it was claimed that the prediction of adverse events or efficacy of treatment on the basis of the dose or serum concentration of an AED is not possible [65]. In contrast, there is evidence that *β* activity induced by barbituric AEDs is missing in patients with severe epileptic conditions [66]. Moreover, high *β* power under clonazepam is significantly correlated with seizure control in children [67]. However, these studies do not directly show predictability of treatment success by EEG characteristics. Several years after the first quantitative examination of AED effects in the EEG, prognostic studies were implemented. Huang and Shen [68] examined the prognostic significance of diazepam-induced EEG changes in epilepsy. After intravenous injection, the effects on scalp EEG were evaluated and correlated with outcome after approximately 3 years in 48 patients. Patients with a visually detectable response in the EEG to diazepam had a good prognosis. This response consisted of an abolition of abnormal activity with emergence of fast activity. Moreover, the diazepam-induced changes in *β* activity were related to the percentage reduction in seizure frequency in these patients. Despite the fact that this work does not provide a quantitative analysis of the EEG responses to AED, it does predict outcome based on the drug responsivity of the EEG. In addition to power changes, it was acknowledged that EEG and magnetoencephalography can aid the prediction of AED efficacy on the basis of spike morphology measurements [69].

Another more recent publication focused on the prognosis of treatment response based on the EEG in patients with idiopathic generalized epilepsies. Patients with a spike-wave onset frequency of > 3.2 Hz became seizure-free with medication after 1–2 years, while those with slower onset frequency did not [70]. The sensitivity in these respects was 72% and the specificity 92%. These remarkable results in a relatively small sample (21 patients) should encourage other researchers to further examine the potential of the EEG in general or under medication (in the sense of pharmaco-EEG) for the prediction of treatment response.

The summarized studies focused on treatment response, but did not include adverse effects in their analysis. One study directly and prospectively related the EEG frequency response to carbamazepine with cognitive adverse effects in children [71]. In 16 children with partial-onset seizures, EEG changes to carbamazepine in relation to baseline were assessed. Neuropsychological changes over a year were predicted by a decrease of the dominant (*α*) rhythm by more than 0.5 Hz. This result emphasizes once again the indicative value of EEG slowing. Some years later, a similar finding was established in adult patients [37]. Test–retest differences before and after 12 weeks of therapy in the EEG were related to cognitive assessment in healthy adults. The posterior peak frequency was decreased after 12 weeks of AED therapy and the strength of the effect varied with the kind of AED. Most strikingly, EEG slowing was correlated with subjectively reported cognitive impairment and with cognitive test performance. The authors furthermore emphasized that the EEG more clearly documented the change, while only single participants exceeded the 95% test–retest interval for the two assessment times in cognitive tests. These results were confirmed and extended in a second publication [53]. In this study, the peak frequency of the EEG rhythm was found to decrease when AED therapy was initiated and to increase when AED therapy was discontinued. Again, this change correlated with cognitive changes and subjective complaints. The authors of this study hypothesized that the frequency changes reflect AED-induced changes in alertness, which, in turn, affects cognitive function [53]. In other words, we could interpret this finding as such that EEG markers correlate with relevant side effects of AEDs.

Nevertheless, we have to conclude that although prognosis of treatment response is clinically very important, the level of evidence is still very low.

## Trends for Pharmaco-EEG: An Undervalued Potential?

Despite the fact that the summarized results sound promising, at the beginning of the new millennium, pharmaco-EEG is not widely applied. Fink [19] regrets that the interest in this type of quantitative science is limited and the enthusiasm is muted, although it would have so many advantages. The potential uses of pharmaco-EEG still need to be developed [17, 18], one of which was addressed in two studies [37, 53]. The authors were interested in the objective measurement of neurotoxicity of AEDs in relation to cognitive side effects of AEDs. Again, the study was based on the many findings that EEG slowing is typical for pharmacological intoxication, specifically with AEDs [71]. At about the same time, technological advances were also introduced in pharmaco-EEG studies. Low-resolution tomography (LORETA) was introduced in order to identify the brain areas mainly involved in psychopharmacological action [72, 73]. Newer publications have not only replicated the wide-band power decrease when AEDs are reduced during intracranial EEG monitoring, but have also used newer EEG features such as Teager energy [74], which was used for seizure detection [72] and was found to decrease in response to AED tapering [32, 33]. Similarly, evolving epileptic networks were believed to be influenced by AED medication changes during pre-surgical evaluation [75]. One study provided direct evidence for the impact of strongly changing serum concentrations of carbamazepine during pre-surgical evaluation [76]. The authors observed that, in the primary epileptogenic area, neuronal complexity loss was inversely related to carbamazepine serum concentrations.

We suggest, furthermore, that the effect of AEDs on electrical brain activity in response to stimulation, such as event-related potentials, deserves more attention. Documentation of the effect of AEDs on the EEG during stimulation is not only an absolute asset for the very specific case of photosensitive epilepsy [77]. In view of the summarized evidence for the direct relationship between behavioral and EEG effects of AEDs, we anticipate that the examination of event-related potentials or modulations of brain rhythms could be a viable method to detect the subclinical effects of AEDs at low doses, such as attention-related activations [52].

Many of these perspectives are still at an early stage of examination. In our view, study of these new developments is worth being extended, specifically for the prediction of treatment response and the prediction of side effects of AEDs.

## Outlook

Care must be taken in the interpretation of pharmaco-EEG [78]. Quantitative EEG offers a wide range of possible interpretations of the signal, which come with a wide range of statistical challenges.

The duration of the recording, which is typically 5 min, should be considered carefully [79]. One obvious aspect that is relevant specifically for patients with epilepsy is the frequency of epileptiform discharges. Quantitative measures extracted from the EEG are affected by the length of the analyzed signal [80] and by pathology, specifically epilepsy [81].

However, recent developments on EEG biomarkers are not yet part of AED pharmaco-EEG. Moreover, recent research in epilepsy has focused on higher frequency ranges, i.e., *γ* (40–80 Hz) and the range of high-frequency oscillations between 80 and 500 Hz. Even if their contribution to pre-surgical evaluation [82, 83] is questionable [84], a critical and systematical appraisal in the pharmaco-EEG context will provide evidence regarding whether inclusion of this frequency range is a valid approach. We also note that pharmaco-EEG with AEDs has been mainly conducted during resting conditions, while its implementation alongside cognitive stimulation might be more useful for the prediction of cognitive side effects.

Most importantly, since interest in pharmaco-EEG has been very limited in the last 10 years, most of the newer drugs have not been subject to pharmaco-EEG studies.

## Conclusions

Pharmaco-EEG in epilepsy has only rarely been conducted under this name, while the use of EEG in epilepsy is a worldwide standard. Older studies have mainly reported frequency responses, while modern quantitative EEG analysis opens up new options that have not been investigated at all. It would be straightforward to incorporate EEG studies in the initial administration of AEDs in patients with epilepsy in order to study short-term effects, and to examine the predictability of treatment response and side effects. However, in order to identify pharmaco-EEG studies as such, the popularity of this technique should be enhanced by indicating the use of EEG in AED studies by explicitly using the term pharmaco-EEG.
