# Correction to: Quantitative Pharmaco-Electroencephalography in Antiepileptic Drug Research

**DOI:** 10.1007/s40263-019-00603-9

**Published:** 2019-02-26

**Authors:** Yvonne Höller, Christoph Helmstaedter, Klaus Lehnertz

**Affiliations:** 1 Department of Neurology, Christian Doppler Medical Centre and Centre for Cognitive Neuroscience, Paracelsus Medical University, Ignaz Harrer Str. 79, 5020 Salzburg, Austria; 2 Present Address: Department of Psychology, University of Akureyri, Norðurslóð 2, 600 Akureyri, Iceland; 3 Department of Epileptology, University of Bonn, Sigmund Freud Straße 25, 53105 Bonn, Germany; 4 Interdisciplinary Center for Complex Systems, University of Bonn, Brühler Straße 7, 53175 Bonn, Germany

## Correction to: CNS Drugs (2018) 32:839–848 10.1007/s40263-018-0557-x

The Open Access license, which previously read:

**Open Access** This article is distributed under the terms of the Creative Commons Attribution-NonCommercial 4.0 International License (http://creativecommons.org/licenses/by-nc/4.0/), which permits any noncommercial use, distribution, and reproduction in any medium, provided you give appropriate credit to the original author(s) and the source, provide a link to the Creative Commons license, and indicate if changes were made.

Should read:

**Open Access** This article is distributed under the terms of the Creative Commons Attribution 4.0 International License (http://creativecommons.org/licenses/by/4.0/), which permits unrestricted use, distribution, and reproduction in any medium, provided you give appropriate credit to the original author(s) and the source, provide a link to the Creative Commons license, and indicate if changes were made.

